# Improved Estimation of Exercise Intensity Thresholds by Combining Dual Non-Invasive Biomarker Concepts: Correlation Properties of Heart Rate Variability and Respiratory Frequency

**DOI:** 10.3390/s23041973

**Published:** 2023-02-10

**Authors:** Bruce Rogers, Marcelle Schaffarczyk, Thomas Gronwald

**Affiliations:** 1 College of Medicine, University of Central Florida, 6850 Lake Nona Boulevard, Orlando, FL 32827-7408, USA; 2 Interdisciplinary Institute of Exercise Science and Sports Medicine, MSH Medical School Hamburg, University of Applied Sciences and Medical University, Am Kaiserkai 1, 20457 Hamburg, Germany

**Keywords:** respiratory frequency, endurance exercise, heart rate variability, intensity distribution

## Abstract

Identifying exercise intensity boundaries has been shown to be important during endurance training for performance enhancement and rehabilitation. Unfortunately, even though surrogate markers show promise when assessed on a group level, substantial deviation from gold standards can be present in each individual. The aim of this study was to evaluate whether combining two surrogate intensity markers improved this agreement. Electrocardiogram (ECG) and gas exchange data were obtained from 21 participants who performed an incremental cycling ramp to exhaustion and evaluated for first (VT1) and second (VT2) ventilatory thresholds, heart rate (HR) variability (HRV), and ECG derived respiratory frequency (EDR). HRV thresholds (HRVT) were based on the non-linear index a1 of a Detrended Fluctuation Analysis (DFA a1) and EDR thresholds (EDRT) upon the second derivative of the sixth-order polynomial of EDR over time. The average of HRVT and EDRT HR was set as the combined threshold (Combo). Mean VT1 was reached at a HR of 141 ± 15, HRVT1 at 152 ± 14 (*p* < 0.001), EDRT1 at 133 ± 12 (*p* < 0.001), and Combo1 at 140 ± 13 (*p* = 0.36) bpm with Pearson’s r of 0.83, 0.78, and 0.84, respectively, for comparisons to VT1. A Bland–Altman analysis showed mean biases of 8.3 ± 7.9, −8.3 ± 9.5, and −1.7 ± 8.3 bpm, respectively. A mean VT2 was reached at a HR of 165 ± 13, HRVT2 at 167 ± 10 (*p* = 0.89), EDRT2 at 164 ± 14 (*p* = 0.36), and Combo2 at 164 ± 13 (*p* = 0.59) bpm with Pearson’s r of 0.58, 0.95, and 0.94, respectively, for comparisons to VT2. A Bland–Altman analysis showed mean biases of −0.3 ± 8.9, −1.0 ± 4.6, and −0.6 ± 4.6 bpm, respectively. Both the DFA a1 and EDR intensity thresholds based on HR taken individually had moderate agreement to targets derived through gas exchange measurements. By combining both non-invasive approaches, there was improved correlation, reduced bias, and limits of agreement to the respective corresponding HRs at VT1 and VT2.

## 1. Introduction

The importance of defining proper exercise intensity boundaries for optimal endurance training outcomes in performance sports and rehabilitation has been increasingly recognized [1,2,3,4,5,6]. Several intensity distribution models have been proposed including a common recommendation that the bulk of training should be spent in a low intensity zone and demonstrate variable amounts both below and above a higher “critical intensity” transition [7,8]. The low intensity zone is classically defined as work rates below the gas exchange threshold (GET), first ventilatory threshold (VT1), or the first lactate threshold (LT1) derived through incremental exercise metabolic cart or lactate testing [8]. The critical intensity, aiming to separate the heavy-to-severe exercise transition, has been referred to by several descriptive concepts such as critical power (CP), maximal lactate steady state, second lactate threshold (LT2), respiratory compensation point (RCP), and second ventilatory threshold (VT2), and is felt to be metabolically unstable and non-sustainable [1,6]. Since both thresholds theoretically require either invasive testing and/or specialized costly equipment, various surrogate markers have been proposed for zone demarcation [9,10,11,12]. Historically, simple zone estimations using heart rate (HR) or HR reserve were suggested but have been shown to be inaccurate [13]. Several alternate non-invasive methods have been developed to improve boundary precision, including those based upon heart rate variability (HRV), the relative perceived effort (RPE), the deoxygenated hemoglobin signal (HHb) from near infrared spectroscopy (NIRS), calculations of CP, and the functional threshold power (FTP) [12,13,14,15]. Considering available methods, only HRV and RPE have been proposed for both low and high intensity threshold identification. In addition, each testing modality has their own particular practical or technical “weaknesses”. For example, HRV may be greatly impacted by signal noise and cardiac arrythmia [16] or NIRS from overlying fat, tissue pigmentation, or probe movement [17,18]. For some modalities such as CP or FTP, repeated maximal voluntary efforts are required that may or may not be achievable or advisable for a given training and recovery schedule. Unfortunately, even though most studies have shown reasonable agreement between many of these methods, with gold standards such as gas exchange or lactate testing on a group basis, differences at the individual level may be significant. Deviations may be physiologically meaningful and could potentially impact the status of fitness, recovery, and rehabilitation [4,5,8]. Given the consequences of exercising at inappropriate work rates based on faulty intensity boundary estimations, the search for ways to enhance non-invasive threshold precision seems worthwhile.

Considering both the presence of individual threshold bias and potential test fallibility, one possible solution is to combine two disparate testing paradigms for an integrated result. This has been suggested by Gaskill [19] for the interpretation of gas exchange results but has not been commonly used with other threshold testing types. Furthermore, it would be ideal to combine methodologies that are measured with similar equipment but represent differing physiologic subsystems in order to reduce cost. HRV-related thresholds (HRVT), obtained through an analysis of the non-linear index a1 of the Detrended Fluctuation Analysis (DFA a1), have shown good reliability in runners, cyclists, cardiac patients, and for both sexes [20,21]. The HRVT1 has been shown to be closely associated with the VT1 or LT1, and the HRVT2 to be associated with the RCP or LT2 in both runners and cyclists but with individual degrees of variation. The DFA a1 reflects autonomic nervous system balance (ANS) and regulation patterns through effects on the cardiac pacemaker cells, resulting in a shift of fractal “correlation” properties as exercise intensity and organismic demands change. At a low exercise intensity, values are typically well correlated (at or above 1.0), decline through the moderately correlated range near the VT1/LT1 (about 0.75), become uncorrelated near the VT2/LT2 (0.5), and decline further into an anticorrelated range above VT2/LT2 work rates (below 0.5). This index is felt to be representative of the “Network” theory of exercise, which is a concept that blends multiple neuromuscular, biochemical, peripheral, and central nervous system (CNS) inputs, leading to an overall assessment of “organismic demand” that is reflected in the short-term scaling exponent of the DFA a1 [20]. Examples of other ANS-related approaches would be other HRV indexes (such as the standard deviation of corrected RR intervals, SDNN, or the standard deviation 1 from a Poincaré plot analysis, SD1) which have been used previously in exercise intensity threshold research [22].

The question arises: What other parameters can be garnered representing another physiologic subsystem using similar measuring equipment consisting of a HR monitoring device with its appropriate software? Since good agreement has been shown between Electrocardiogram (ECG)-derived respiratory frequency (EDR) and gas exchange values [23], it may be possible to obtain threshold boundaries via an analysis of the EDR. Both conventional gas exchange [24] and EDR derived respiratory rates [25] have been successfully used for VT1/VT2 identification. One calculation technique relies upon the detection of two areas of maximal acceleration of the respiratory frequency (RF) over time during an incremental ramp to exhaustion, which correspond to the VT1 and VT2. Cross et al. [24] showed that the first RF threshold agreed with the VT1 and the second threshold with the VT2 in a group of recreational cyclists. Their method consisted of first plotting the 6th order polynomial spline of the RF over time during an incremental ramp to exhaustion. A calculation yielding the two instances of the second derivative maxima (curve acceleration points) of this sixth-order trendline is then obtained that corresponds to the first and second thresholds. Differing from the ANS-related DFA a1 metric, exercise-induced RF change is felt to be due to a combination of afferent muscle signaling and a concept termed “central command” [26,27]. Central command refers to RF control via inputs from the periaqueductal gray area, supplementary motor area, the premotor area, and the prefrontal cortex. Hence, both the DFA a1 and RF could be measured with the same wearable ECG device and appropriate software but may reflect different underlying physiological foundations.

The aim of the present study was to answer two questions: (1) can the ECG-derived RF threshold (EDRT1/2) based on HR be used as a surrogate measure for gas exchange thresholds? and (2) does averaging the HRVT1/2 based on HR with the EDRT1/2 result in an improved correlation and/or agreement with the gas exchange-derived thresholds as a gold standard? Considering practical aspects for training guidelines in sports and rehabilitation, only HR measurements will be compared, given the excellent agreement between the HR and VO_2_ relationship during exercise [28].

## 2. Methods

### 2.1. Participants

A diverse group of 21 physically active participants were enlisted through the local community (men: N = 12, women: N = 9). The included participant data were based on a validation study of the Movesense Medical ECG sensor and Kubios HRV software to determine the RF [22]. The offer for enrollment applied to women and men of any fitness level without previous medical history, current medications, or recent illness, with a minimum age of 18 years. Participants were asked to abstain from caffeine, alcohol, tobacco, and strenuous exercise for 24 h before testing. Written informed consent was obtained from each participant. One participant with varying degrees of atrioventricular block was excluded from analysis. Ethical approval for the study was obtained through the University of Hamburg, Department of Psychology and Movement Science, Germany (reference no.: 2021_400) and was in accordance with the principles of the Declaration of Helsinki.

Exercise Protocol and Data Recording

An incremental ramp protocol until volitional exhaustion was performed on a mechanically braked cycle (Ergoselect 4 SN, Ergoline GmbG, Bitz, Germany) by all 21 participants. The testing procedure was as follows: an initial warm-up of three minutes with a starting workload of 50 watts was begun, immediately followed by the incremental ramp, by increasing 1 watt every 3.6 s (equivalent to 50 watts/3 min). The exercise ramp was concluded when the participant could not maintain a cadence of 60 rpm or when they reached subjective exhaustion or a heart rate > 90% of the maximum predicted heart rate, or respiratory exchange ratio > 1.1. ECG recordings were taken continuously with the Movesense Medical sensor (firmware version 2.0.99) single-channel ECG with a chest belt (Movesense, Vantaa, Finland; sampling rate: 512 Hz; app software: Movesense Showcase version 1.0.9). Placement of the chest belt device was just below the pectoral muscles and centered at the sternum. Gas exchange kinetics were recorded with a metabolic analyzer (Quark CPET, module A-67-100-02, Cosmed, Rome, Italy; desktop software: Omnia version 1.6.5). Maximum oxygen uptake (VO_2MAX_) and maximum HR (HR_MAX_) were defined as the maximal 20 s average VO_2_ and HR over the exercise portion of the test.

### 2.2. Data Processing

ECG tracings were recorded via an iPhone, converted into .csv files, and then processed using Kubios HRV Premium Software version 3.5 (Biosignal Analysis and Medical Imaging Group, Department of Physics, University of Kuopio, Kuopio, Finland). Visual inspection of the entire test recording was performed to determine the sample quality, noise, arrhythmia, and missed beat artifact. Preprocessing settings were set to the default values, including the RR detrending method, which was kept at “smoothness priors” (Lambda = 500). The RR series was then corrected using the Kubios HRV Premium “automatic method”. DFA a1-related “short term fluctuations” was set to 4 ≤ n ≤ 16 beats in the software preferences. For the RF calculation, the measurement window width was set to 30 s, with a recalculation grid interval performed every 1 s [23]. For the DFA a1 calculation, the window width was set to 120 s, with a recalculation grid interval performed every 5 s [10]. Data sets with artifacts > 8% were excluded from analysis for EDR thresholds and >5% for the DFA a1 thresholds [16].

### 2.3. Calculation of VTs

VO_2_, VCO_2_, PetO_2_, PetCO_2_, Ve/VO_2_, Ve/VCO_2_, and HR were imported into Microsoft Excel 365 for analysis via the Quark CPET. To maximize the precision of the VT1 via gas exchange, an evaluation was performed according to the triple detection method consisting of V slope, Ve/VO_2_, and excess CO_2_ [18] as well as the PetO_2_ nadir [29]. Results were reviewed independently by two investigators. VT2 was determined via the method of RCP deflection of PetCO_2_ [29]. The HR at both the VT1 and VT2 was set as the HR at each ramp-related point in time.

### 2.4. Calculation of the HRVTs

**Figure 1:**
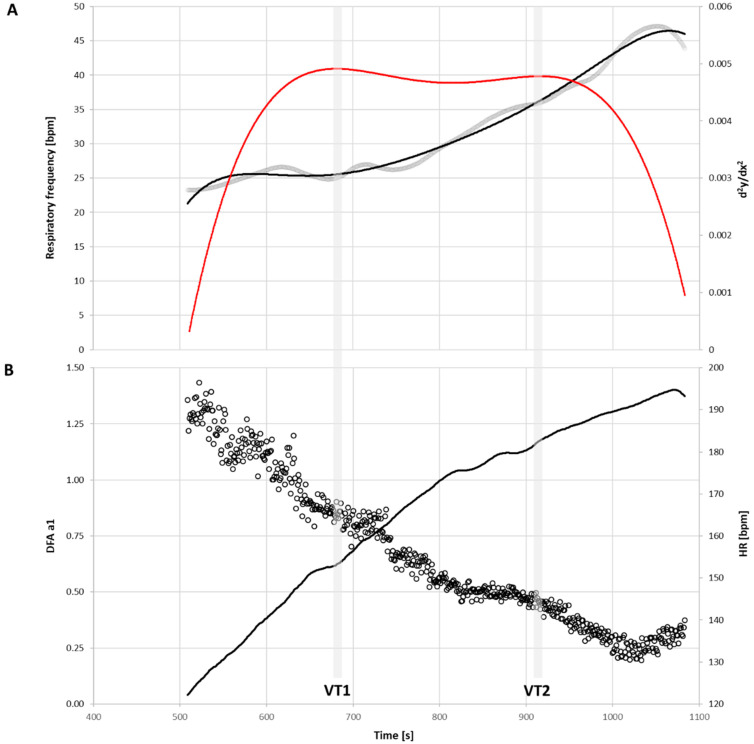
A 38-year-old male with a VO_2MAX_ of 43.1 mL/kg/min performing the incremental cycling ramp to exhaustion; vertical gray bars represent VT1 and VT2 respectively. (**A**) RF (bpm, gray line), the sixth order polynomial spline (black line) and the second derivative of the polynomial spline (d^2^y/dx^2^, red line). (**B**) DFA a1 over time (open circles) and HR (bpm, black line).

The following procedure was used to indicate at what HR the DFA a1 would cross a value of either 0.75 (HRVT1) or 0.5 (HRVT2): plotting of the DFA a1 vs. HR was performed. Inspection of the DFA a1 relationship with HR generally showed a reverse sigmoidal curve, with a stable area above 1.0 at the moderate exercise intensity domain, a rapid, a near linear drop reaching below 0.5 at the heavy and severe exercise intensity domain, and then flattening to a nadir (Figure 1). Linear regression was performed on the subset of data consisting of the rapid, near straight-line drop from values close to 1.0 (well correlated) to approximately 0.5 (uncorrelated), or below if the values continued in a non-deviating fashion. The HR where the DFA a1 reached either 0.75 or 0.5 was calculated based on the regression equation from that linear section [10,11].

### 2.5. Calculation of the EDRTs

Derivation of EDR-related thresholds was based on the method proposed by Cross et al. [24]. EDR was extracted from Kubios Premium software for each participant ramp test as previously outlined and plotted over time [23]. A 6th-order polynomial spline was fitted to the span of data after the warm-up, ending at the ramp termination. The resulting equation was used to calculate the RF data points over the above data span. The second derivative of this data series was then calculated and plotted over time to confirm that 2 distinct points of maxima appeared over the time series. If only one local maximum was noted, the ramp measurement start time was moved upward by 30 s and the termination was extended by 5 s. The time of the first maximum was set as the EDRT1 and the time of second maximum as EDRT2 (Figure 1). Corresponding HR derived from the matching EDRT measurement window was set as the threshold-related HR.

### 2.6. Calculation of HRVT and EDRT Combination

The values for the HRVT and EDRT HR for a particular threshold were averaged to create a combined (Combo) value (HRVT1 + EDRT1)/2 = Combo1, (HRVT2 + EDRT2)/2 = Combo2. For the purpose of more widespread use of this technique using Microsoft 365 only, a worksheet example of the EDRT calculation is presented in the Supplementary File S1.

## 3. Statistics

The normal distribution of data was checked using a visual inspection of the data histograms and via Shapiro–Wilk testing. Descriptive statistical analysis was performed using Microsoft Excel 365 for the calculation of means and standard deviations (SD). The agreement of the threshold data was assessed via linear regression, Pearson’s r correlation coefficient, standard error of estimate (SEE), and Bland–Altman plots with limits of agreement (LoA). The size of Pearson’s r correlations was evaluated as follows: 0.3 ≤ r < 0.5 low, 0.6 ≤ r < 0.8 moderate, and r ≥ 0.8 high [30]. Intraclass correlation coefficient (ICC_3,1_) with 95% confidence intervals (CI) was used to evaluate the VT, HRVT, EDRT, and Combo intergroup reliability, and was further categorized with results being <0.40 as poor, 0.40 to 0.59 as fair, 0.60 to 0.74 as good, and 0.75 to 1.00 as excellent [31]. The Bland–Altman mean differences for data comparisons were expressed as the absolute differences in HR as beats per minute [32]. Inspection of the distribution of the mean differences in the Bland–Altman analysis was performed to confirm normality and if proportional bias was detected, a regression-based calculation of mean differences and LoA were presented [33]. Paired *t*-testing was used for the comparison of gas exchange thresholds (VT1/2) vs. each surrogate modality (HRVT1/2, EDRT1/2, Combo1/2). For all tests, the statistical significance was accepted as *p* ≤ 0.05. Analytical statistics were performed using Microsoft Excel 365 with Real Statistics Resource Pack software (Release 7.6, copyright 2013–2021, Charles Zaiontz, www.real-statistics.com) and Analyse-it software (Leeds, UK, Version 6.01).

## 4. Results

Means with standard deviations for age, body weight, height, maximum HR, and VO_2MAX_ achieved during the incremental test are presented in Table 1.

### EDRT, HRVT and Combo Agreement to VTs

Due to artifacts of 5 to 8%, four cases were excluded from HRVT assessment [16]. All artifacts were premature atrial or ventricular complexes. Individual participant results for VT1, HRVT1, EDRT1, Combo1, VT2, HRVT2, EDRT2, and Combo2, as well as mean and standard deviations, are presented in Table 2. The mean HR values for the HRVT1 (152 ± 14 bpm) and EDRT1 (133 ± 12 bpm, *p* < 0.001) did differ from those of the VT1 HR (141 ± 15 bpm, *p* < 0.001) on paired t testing, but the Combo1 HR (140 ± 13 bpm, *p* = 0.36) was not significantly different than the VT1 HR. In the case of the VT2 comparisons, the HRVT2 (167 ± 10 bpm, *p* = 0.89), EDRT2 (164 ± 14 bpm, *p* = 0.36), and Combo2 (164 ± 13 bpm, *p* = 0.59) were not significantly different than the VT2 HR (165 ± 13 bpm). An excellent degree of reliability was seen between all measures (N = 16, grouped VT, HRVT, EDRT, Combo), with an average ICC_3,1_ of 0.78 (CI: 0.61–0.91, *p* = 0.05) for the first threshold and 0.75 (CI: 0.56–0.89, *p* = 0.05) for the second threshold data.

**Figure 2:**
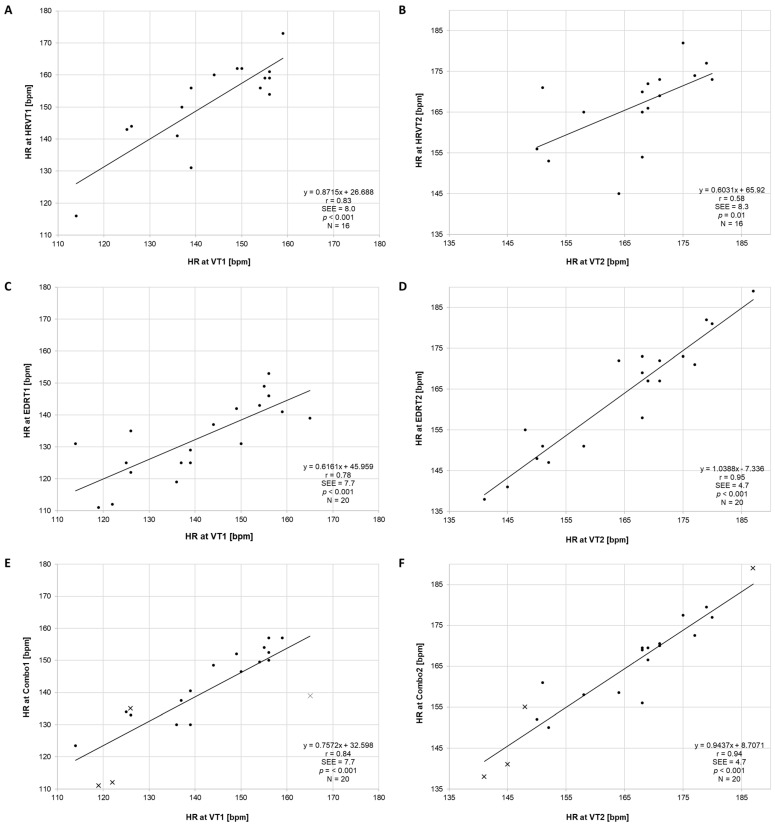
Linear regression of HR for VT1 vs. HRVT1 (**A**), VT2 vs. HRVT2 (**B**), VT1 vs. EDRT1 (**C**), VT2 vs. EDRT2 (**D**), VT1 vs. Combo1 (**E**), and VT2 vs. Combo2 (**F**) with standard error of estimate (SEE), Pearson’s r, and total tests compared (N). Combo values based on only a single threshold modality (EDRT) are represented as “×”.

**Figure 3:**
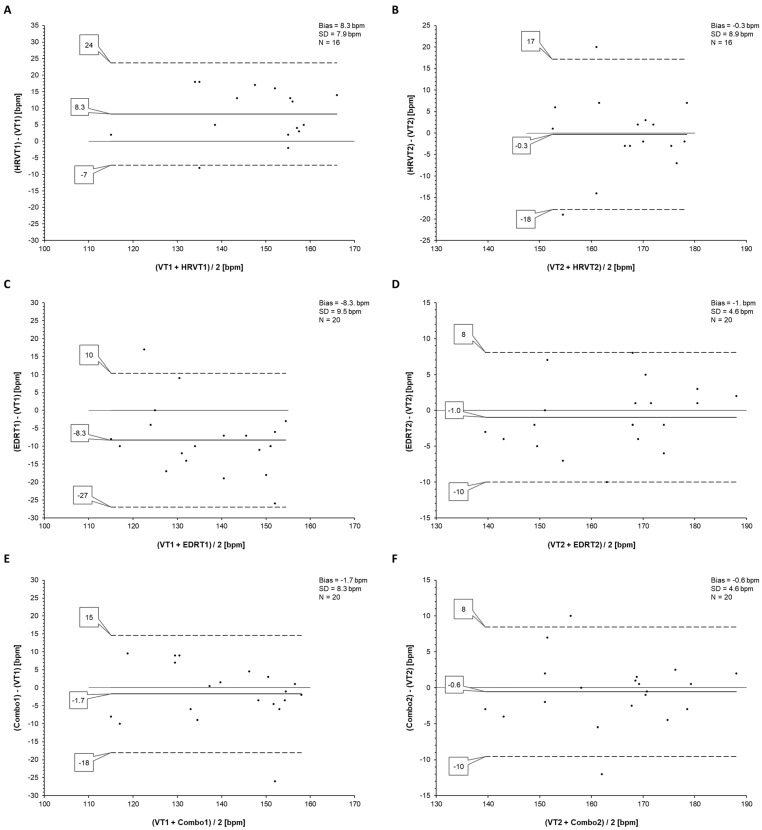
Bland–Altman analysis for VT1 vs. HRVT1 (**A**), VT2 vs. HRVT2 (**B**), VT1 vs. EDRT1 (**C**), VT2 vs. EDRT2 (**D**), VT1 vs. Combo1 (**E**), and VT2 vs. Combo2 (**F**) with bias, standard deviation (SD), and total tests compared (N). Center line in each plot represents the mean difference between each paired value; the top and bottom lines are 1.96 SD from the mean difference.

The regression plots for the HRVT, EDRT, and Combo results are shown in Figure 2. The Bland–Altman analysis for these parameters is shown in Figure 3. Of particular note is the bias and LoA present between VT1 and the HRVT1 HR (8.3 bpm, −7 to 24 bpm); EDRT1 (−8.3 bpm, −27 to 10 bpm) that was markedly improved with the Combo1 HR (−1.7 bpm, −18 to 15 bpm).

## 5. Discussion

In past reports, each single surrogate modality (e.g., HRVT, EDRT) showed reasonable agreement to the “gold standard” group averages but variable degrees of deviation at the individual level [20,24,25]. Although validation with established threshold methodology is the scientific standard introducing novel approaches, it should be understood that other concepts may be based on various physiological mechanisms (e.g., systemic approaches related to ANS or CNS regulation) that may not exactly match with metabolic approaches. However, since training regimes often rely upon proper intensity zone demarcation, failure to locate thresholds precisely could undermine sought after benefits, leading to undesirable effects. With these thoughts in mind, efforts to improve these boundary demarcations on an individualized basis appear worthwhile. The current data suggest that combining both the HRV- and EDR-related thresholds into a single average value results in better correlation and agreement with gas exchange-derived thresholds. Additionally, though the main purpose of this study was to evaluate a combined approach for surrogate threshold determination, an exploration into the previously noted EDRT agreement with VT1/2 was necessary, since so few supporting publications were available. As previously reported, a single index correlation to either VT1/LT1 or VT2/LT2 was in the moderate range, with substantial LoAs [20,21,24,25,34,35]. HRVT1/2-related HRs displayed correlations and LoA similar to the reported values (HRVT1: r = 0.83, bias = 8.3 bpm, LoA = −7 to 24 bpm, HRVT2: r = 0.58, bias = −0.3 bpm, LoA = −18 to 17 bpm). The literature regarding RF-derived thresholds or EDRT agreement with gas exchange-derived thresholds is sparse, with little in the way of HR-related information. EDRT validation results from the present study were somewhat similar to the DFA a1-derived HRVTs (EDRT1: r = 0.78, bias = −8.3 bpm, LoA = −27 to 10 bpm, EDRT2: r = 0.95, bias = −1.0 bpm, LoA = −10 to 8 bpm), with a main aspect being the similar absolute bias present for the first threshold, but in the opposite direction. Additionally, the mean HRVT1 and EDRT1 HR were both significantly different than the VT1 HR (*p* < 0.001). However, by combining the HR values obtained from each technique, the resultant Combo1-related correlations, bias, and LoA to VT1 were moved into improved ranges (Combo1: r = 0.84, bias = −1.7 bpm, LoA = −18 to 15 bpm) with a similar mean group value (VT1 HR =140 bpm, Combo1 HR = 141 bpm, *p* = 0.36). The second threshold modalities all had similar mean values confirmed via paired t testing. However, the moderate correlation of the HRVT2 HR (r = 0.58, bias = −0.3 bpm, LoA = −18 to 17 bpm) was moved to a high level with decreased LoA (Combo2 r = 0.94, bias = −0.6 bpm, LoA −10 to 8 bpm) by combining it with the EDRT HR. The already high EDRT2 HR correlation (r = 0.95), along with its low bias and LoAs (bias = −1.0 bpm, LoA −10 to 8 bpm), were minimally altered via a combination with the HRVT2 HR. Hence, our data show excellent concordance of the EDRT2 with the VT2 as a single measure without any disadvantage to combining it with the HRVT2 HR in the present sample. Furthermore, in cases where one modality failed due to technical reasons (e.g., HRV artifact excluding HRVT measurement recommendations), the remaining method appears to still provide good agreement to gas exchange-derived thresholds. Although we did not encounter any cases where EDRT was unobtainable, previous reports have indicated that this is certainly possible [24,25].

Each surrogate marker utilized in this study used the same monitoring device but reflect different underlying aspects of physiology. The DFA a1 is a HRV index with a wide dynamic range through all exercise intensities that is dependent on ANS balance and regulation pattern with resultant effects on the cardiac pacemaker cells [20,36]. As exercise intensity rises, the parasympathetic/sympathetic balance and antagonistic behavior shifts, leading to changes in the fractal correlation properties of HRV. However, an alternate approach to explaining this metric is through the understanding of something called the “network theory of exercise physiology” [37]. This refers to diverse inputs from neuromuscular, biochemical, peripheral, and CNS sources leading to an overall concept of “organismic demand” that is reflected in HRV indexes such as the DFA a1. This index has shown utility in threshold determination, fatigue monitoring, daily directed training, and even in atypical exercise scenarios such as eccentric cycling [20]. There are inherent limitations in this approach as it depends on a precise RR interval measurement along with the issues involving HRV artifact correction [20]. With respect to the RF control, fundamental mechanisms are also partly under “central command”(periaqueductal gray area), with other factors also included, most notably muscle afferent input [27]. Additionally, the exact CNS centers responsible for RR interval timing (HRV) [38,39,40] and RF may or may not overlap. Cardiac vagal fibers originating in the nucleus ambiguus are predominantly responsible for vagal HRV effects; however, significant crosstalk with respiratory control mechanisms may also be present in the peripheral vagal ganglia [40]. Although there may be some commonality in the CNS control [26,40], a look at DFA a1 and RF behavior does reveal marked differences (Figure 1). During the incremental exercise ramp, there is a linear decline in DFA a1 starting well before VT1 that continues past VT2 in most individuals. In contrast, the RF displays a change in acceleration (the RF second derivatives) at VT1 and VT2. Therefore, the RF is characterized as having “breakpoint” behavior at the EDRT1/2 with DFA a1 reaching pre-defined levels of correlation properties at the HRVT1/2 with the current methodological approach.

An interesting finding of the present report was that the mean bias for the HRVT1 HR was higher and the EDRT1 HR lower-than-gold-standard gas exchange-derived values. However, when these single source values were combined, the resultant Combo1 HR mean bias was virtually eliminated, with similar mean HR values to the VT1 HR, along with improved correlation. Whether this reflects self-correcting differences in marker determination through different underlying physiologic control processes (ANS regulation patterns vs. RF control mechanisms within the CNS), fundamental issues in index measurement (methodological bias in one method counterbalanced by the other), and/or random chance, is unclear. Further exploration of this question is recommended.

## 6. Limitations

Several physiologic and technical shortcomings are possible for any HRV-related measurement. The occurrence of artifacts from either ECG signal noise and/or cardiac arrythmia could lead to bias in the DFA a1 estimation with mild effects at 3% but more substantial effects above 5% [16]. Also, the effects of Kubios HRV software artifact correction on the EDR estimation is unknown and should be examined in the future. In this report, it was decided to “relax” the 5% artifact standard usually used in the case of a HRV analysis to include four additional cases of EDRT. In the four cases seen with the artifact at 5 to 8%, all artifacts were premature atrial or ventricular complexes and did not appear to affect the RF curve [23]. Perhaps this is due to the EDR being based on the ECG voltage rather than RR interval length. However, effects of substantially higher degrees of noise or arrythmia may indeed lead to EDR curve distortion. Another potential question is whether the HRVT/EDRT results could have shown better agreement with gas exchange data if the HR recording device position was optimized for best R wave peak amplitude. It has been shown that ECG lead placement can affect DFA a1 results, presumably through changes in waveform morphology and the signal-to-noise ratio [41]. In a similar fashion, the EDR also depends on R wave amplitude change, illustrating the importance of achieving the best ECG waveform before performing testing. Results of both the DFA a1 and EDR were obtained with Kubios HRV Premium software; the utilization of alternate software systems using dissimilar methodology (preprocessing, artifact correction, EDR) may yield different results. Looking at this study’s participant population, there was high diversity regarding VO_2MAX_, sex, and age. Between-method agreement may have been different across more homogenous populations. Finally, both RF and DFA a1 can be affected by prior exercise, fatigue, as well as other factors which should be considered before testing [20,26,27].

A general concern when deriving exercise intensity thresholds through alternative procedures is the consequence of inaccurate results. An argument can be made to rely on threshold data that are only obtained from gold standard testing approaches. However, since most users do not have easy access to gas exchange devices or lactate testing for various reasons, a more pragmatic approach seems reasonable. In addition, even gas exchange and lactate testing can lead to erroneous results for various reasons, and these metrics may not fully correlate with each other for the purpose of intensity threshold demarcation [42,43,44,45,46]. In the current study, both the Movesense Medical ECG recording device and Kubios HRV Premium software are readily available, allowing athletes, coaches, and rehabilitation centers to potentially take advantage of the proposed technique. A supplementary step by step guide is included to demonstrate how consumer end users can reproduce the EDRT methodology with simple Microsoft Excel calculations.

## 7. Conclusions

Both the DFA a1 and EDR HR-based intensity thresholds taken individually had moderate agreement with targets derived through gas exchange. However, when the two surrogate markers were averaged together, there was improved correlation, reduced bias, and LoAs to the respective corresponding HRs at VT1 and VT2. Furthermore, utilizing two distinct testing components allows for a lower chance of testing assessment failure. Improving the precision of non-invasive exercise intensity boundaries could lead to superior athletic training outcomes, more efficient recovery, and rehabilitation safely. Further exploration into the use of intensity zone demarcation through combining surrogate internal load markers is recommended.

## Figures and Tables

**Table 1:** Demographic data of all included participants (N = 20), with sex, age, height, body weight, maximum heart rate (HR_MAX_), and VO_2MAX_ seen during the incremental test. Mean ± standard deviations (SD) listed.

Sex	Age [Years]	Height [cm]	Body Weight [kg]	HR_MAX_ [bpm]	VO_2MAX_ [mL/kg/min]
M (N = 12)	42.8 (±12.9)	178.2 (±7.8)	83.3 (±13.3)	176.3 (±15.4)	41.4 (±8.8)
F (N = 8)	35.8 (±10.9)	169.4 (±4.4)	65.6 (±9.8)	174.4 (±6.3)	40.2 (±4.9)

**Table 2:** Individual results for VT1, HRVT1, EDRT1, Combo1, VT2, HRVT2, EDRT2, and Combo2 with participant numbers during the incremental test. Mean, standard deviations (SD), Pearson’s r (vs. ventilatory derived thresholds), *p* value for t testing (vs. ventilatory derived thresholds) in last rows.

	VT1 HR	HRVT1 HR	EDRT1 HR	Combo1 HR	VT2 HR	HRVT2 HR	EDRT2 HR	Combo2 HR
	N = 20	N = 16	N = 20	N = 20	N = 20	N = 16	N = 20	N = 20
	156	161	153	157	179	177	182	180
	150	162	131	147	169	172	167	170
	125	143	125	134	152	153	147	150
	137	150	125	138	151	171	151	161
	156	159	146	153	171	173	167	170
	165	-	139	139	187	-	189	189
	119	-	111	111	145	-	141	141
	159	173	141	157	175	182	173	178
	126	-	135	135	148	-	155	155
	122	-	112	112	141	-	138	138
	139	131	129	130	158	165	151	158
	154	156	143	150	180	173	181	177
	126	144	122	133	150	156	148	152
	155	159	149	154	177	174	171	173
	136	141	119	130	168	154	158	156
	139	156	125	141	169	166	167	167
	144	160	137	149	168	170	169	170
	156	154	146	150	168	165	173	169
	149	162	142	152	171	169	172	171
	114	116	131	124	164	145	172	159
**Mean ± SD**	141 ± 15	152 ± 14	133 ± 12	140 ± 13	165 ± 13	167 ± 10	164 ± 14	164 ± 13
**r**	-	0.83	0.78	0.84	**-**	0.58	0.95	0.94
***p***	-	<0.001	<0.001	0.36	**-**	0.89	0.36	0.59

## Data Availability

The raw data supporting the conclusions of this article will be made available by the authors, without undue reservation.

## Supplementary Materials

The following supporting information can be downloaded at: https://www.mdpi.com/article/10.3390/s23041973/s1.
