# Supplementary material for: Improved Estimation of Exercise Intensity Thresholds by Combining Dual Non-Invasive Biomarker Concepts: Correlation Properties of Heart Rate Variability and Respiratory Frequency
Source: Sensors (Basel). 2023 Feb 10;23(4):1973. doi: 10.3390/s23041973 (PMC9967516; doi:10.3390/s23041973)
Supplement: Supplementary file 1 [file sensors-23-01973-s001.zip › sensors-2169413-supplementary.pdf]

Supplementary material:

Step by step method to determine EDRT1 and 2 from Kubios HRV software EDR data

Window width should be 30 seconds and grid interval (recalculation) every 1 second (Kubios time varying preferences). Open the .csv file and save as .xlsx. Extract EDR from ECG recording of incremental exercise ramp (Kubios – file – Save as – save as .csv).

Open the saved .csv and save that as an xlsx. Copy columns containing time, mean HR and respiratory rate to a new tab. Note: Kubios calculates respiratory rate as Hz which will need to be converted to bpm.

Create a new column containing respiratory rate as breaths per min (column D x 60):

E3 | fx | =PRODUCT(D3,60)

	A	B	C	D	E	F
1		Time	Mean H	RESP		
2		(hh:mm::	(1/min)	(Hz)		
3		00:00:15	99.3551	0.2299	13.794	
4		00:00:16	99.5415	0.2304		
5		00:00:17	99.6297	0.2309		
6		00:00:18	99.627	0.2315		
7		00:00:19	99.7403	0.232		
8		00:00:20	100.0717	0.2325		
[illegible]		[illegible]	[illegible]	[illegible]		

Extend the new column to the end:

A	B	C	D	E
	Time	Mean H	RESP	Resp rate
	(hh:mm::	(1/min)	(Hz)	bpm
	00:00:15	99.3551	0.2299	13.794
	00:00:16	99.5415	0.2304	13.824
	00:00:17	99.6297	0.2309	13.854
	00:00:18	99.627	0.2315	13.89
	00:00:19	99.7403	0.232	13.92
	00:00:20	100.0717	0.2325	13.95
	00:00:21	100.3069	0.233	13.98
	00:00:22	100.7791	0.2335	14.01
	00:00:23	101.1489	0.234	14.04
	00:00:24	101.9353	0.2345	14.07
	00:00:25	102.3197	0.235	14.1
	00:00:26	102.8352	0.2355	14.13
	00:00:27	103.1006	0.2361	14.166
	00:00:28	103.4829	0.2366	14.196
	00:00:29	103.6156	0.2372	14.232
	00:00:30	103.7215	0.2379	14.274

Populate column A as time in seconds:

A	B	C	D	E
	Time	Mean H	RESP	Resp rate
	(hh:mm::	(1/min)	(Hz)	bpm
15	00:00:15	99.3551	0.2299	13.794
16	00:00:16	99.5415	0.2304	13.824
17	00:00:17	99.6297	0.2309	13.854
18	00:00:18	99.627	0.2315	13.89
19	00:00:19	99.7403	0.232	13.92
20	00:00:20	100.0717	0.2325	13.95
21	00:00:21	100.3069	0.233	13.98
22	00:00:22	100.7791	0.2335	14.01
[illegible]	[illegible]	[illegible]	[illegible]	[illegible]

Plot Time (column A) vs respiratory rate (column E):

	A	B	C	D	E
1		Time	Mean H	RESP	Resp rate
2		(hh:mm::	(1/min)	(Hz)	bpm
3	15	00:00:15	99.3551	0.2299	13.794
4	16	00:00:16	99.5415	0.2304	13.824
5	17	00:00:17	99.6297	0.2309	13.854
6	18	00:00:18	99.627	0.2315	13.89
7	19	00:00:19	99.7403	0.232	13.92
8	20	00:00:20	100.0717	0.2325	13.95
9	21	00:00:21	100.3069	0.233	13.98
10	22	00:00:22	100.7791	0.2335	14.01
11	23	00:00:23	101.1489	0.234	14.04
12	24	00:00:24	101.9353	0.2345	14.07
13	25	00:00:25	102.3197	0.235	14.1
14	26	00:00:26	102.8352	0.2355	14.13
15	27	00:00:27	103.1006	0.2361	14.166
16	28	00:00:28	103.4829	0.2366	14.196
17	29	00:00:29	103.6156	0.2372	14.232
18	30	00:00:30	103.7215	0.2379	14.274
19	31	00:00:31	103.8243	0.2385	14.31
20	32	00:00:32	104.0068	0.2392	14.352
21	33	00:00:33	104.1814	0.2399	14.394
22	34	00:00:34	104.1933	0.2406	14.436
23	35	00:00:35	104.229	0.2414	14.484

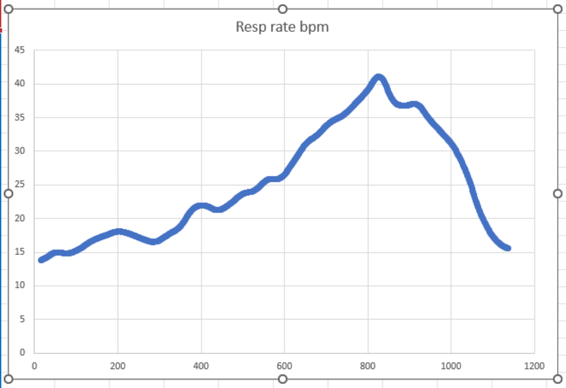

Trim the data to include only the ramp (remove rows at the beginning warmup or end cooldown):

	A	B	C	D	E
1		Time	Mean H	RESP	Resp rate
2		(hh:mm::	(1/min)	(Hz)	bpm
3	311	00:05:11	121.7236	0.2869	17.214
4	312	00:05:12	121.9762	0.2876	17.256
5	313	00:05:13	122.177	0.2883	17.298
6	314	00:05:14	122.5045	0.289	17.34
7	315	00:05:15	122.7572	0.2896	17.376
8	316	00:05:16	123.0845	0.2903	17.418
9	317	00:05:17	123.3655	0.291	17.46
10	318	00:05:18	123.688	0.2916	17.496
11	319	00:05:19	123.9718	0.2923	17.538
12	320	00:05:20	124.1961	0.2929	17.574
13	321	00:05:21	124.3217	0.2935	17.61
14	322	00:05:22	124.4484	0.2942	17.652
15	323	00:05:23	124.5145	0.2948	17.688
16	324	00:05:24	124.5746	0.2954	17.724
17	325	00:05:25	124.6609	0.296	17.76
18	326	00:05:26	124.8015	0.2966	17.796
19	327	00:05:27	124.9284	0.2973	17.838
20	328	00:05:28	125.0535	0.2978	17.868
21	329	00:05:29	125.2249	0.2984	17.904
22	330	00:05:30	125.3898	0.299	17.94
23	331	00:05:31	125.5344	0.2996	17.976

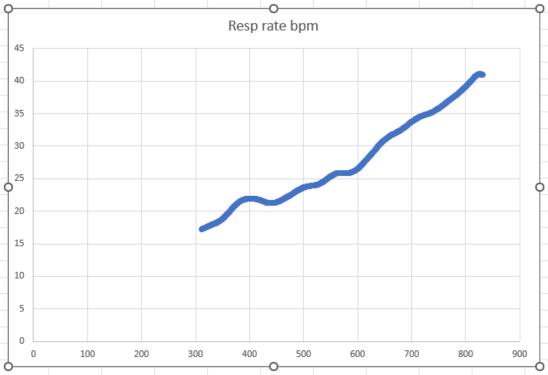

Remove row 2 since it does not contain any data.

Create a trendline – 6th order polynomial:

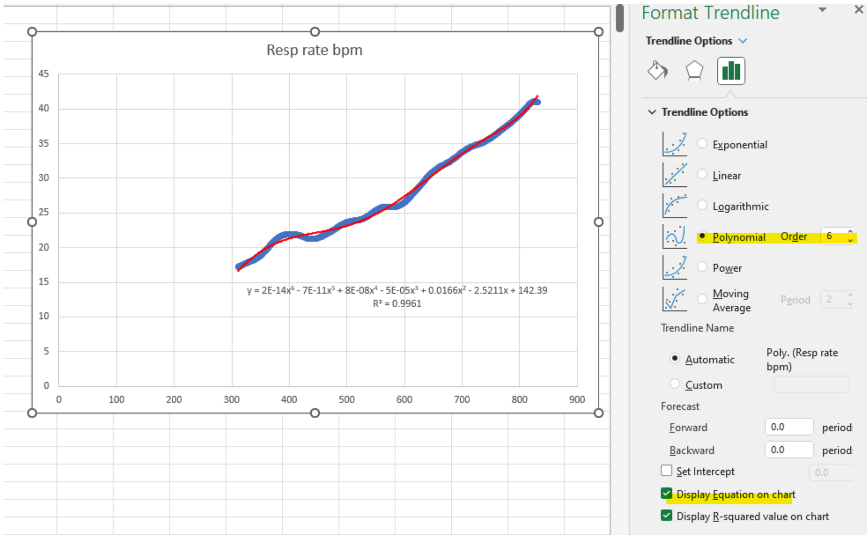

Format the equation - "Trendline Label" (important, the default equation can't be used in the rows):

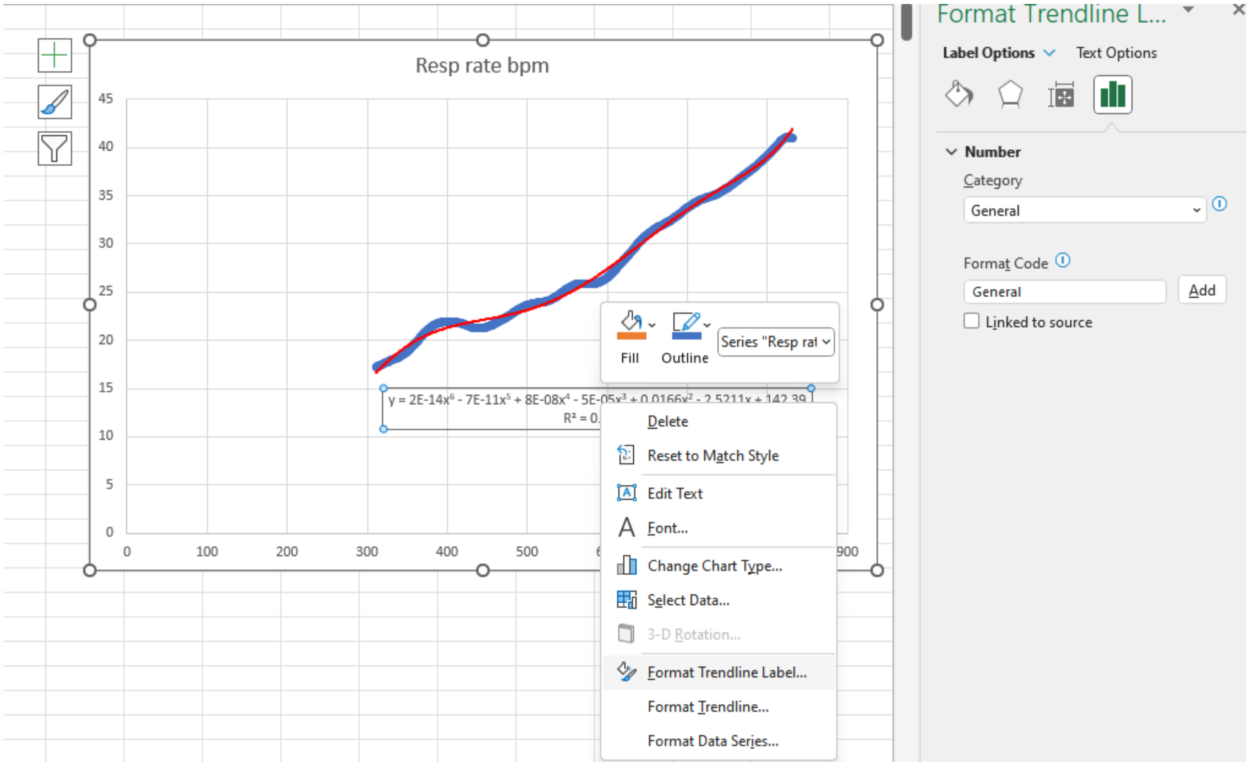

Format as a number with 20 decimals:

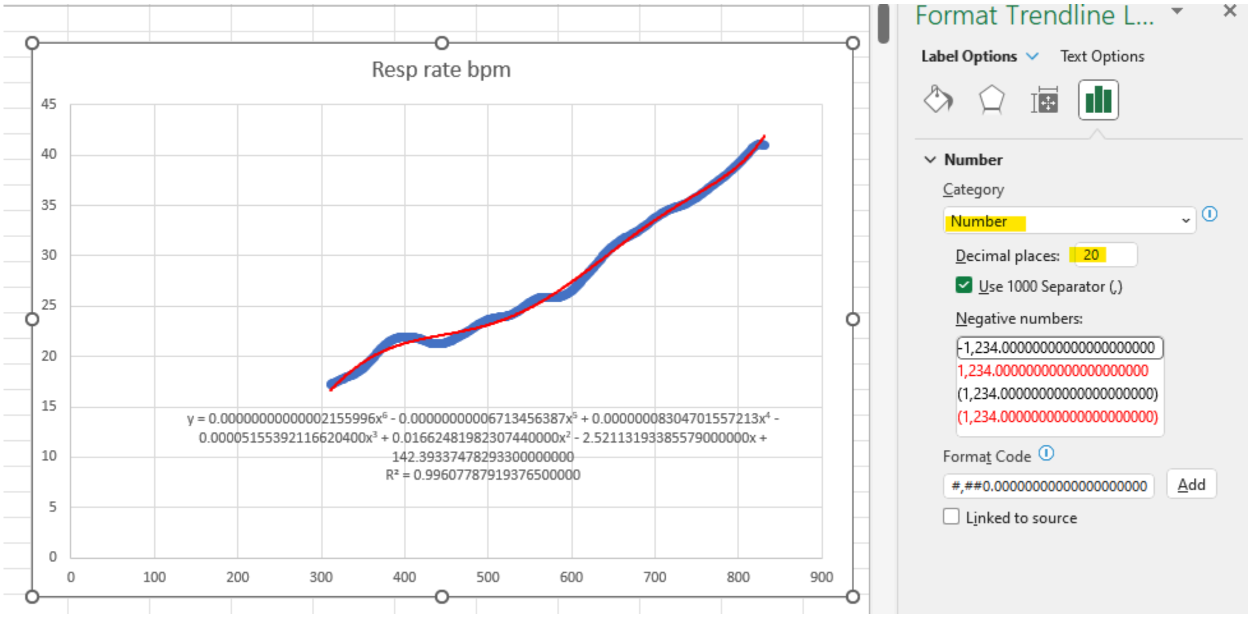

Copy the displayed equation to MS Word:

y = 0.0000000000000002155996x^6 - 0.0000000000006713456387x^5 + 0.000000008304701557213x^4 - 0.00005155392116620400x^3 + 0.01662481982307440000x^2 - 2.52113193385579000000x + 142.39337478293300000000

Find and replace as follows:

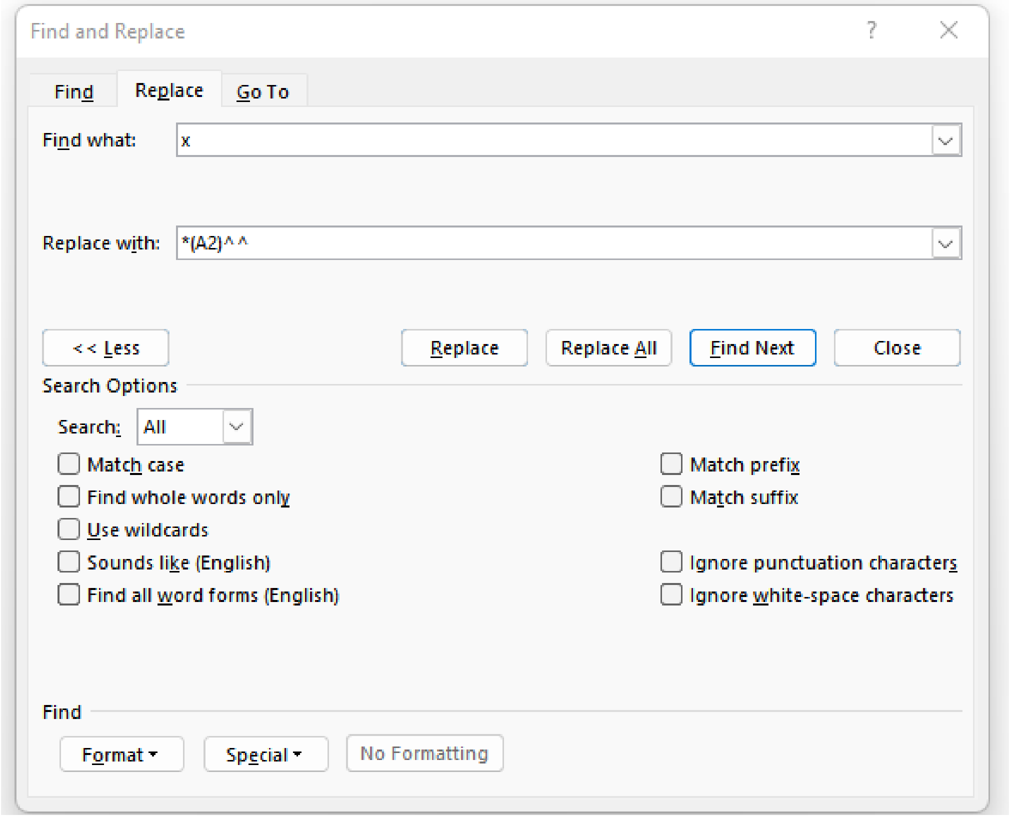

Equation now looks like this:

y = 0.00000000000002155996*(A2)^6 - 0.00000000006713456387*(A2)^5 + 0.00000008304701557213*(A2)^4 - 0.00005155392116620400*(A2)^3 + 0.01662481982307440000*(A2)^2 - 2.52113193385579000000*(A2)^ + 142.39337478293300000000

Remove the y and the ^ from the last (A2)^ notation:

= 0.00000000000002155996*(A2)^6 - 0.00000000006713456387*(A2)^5 + 0.00000008304701557213*(A2)^4 - 0.00005155392116620400*(A2)^3 + 0.01662481982307440000*(A2)^2 - 2.52113193385579000000*(A2) + 142.39337478293300000000

Copy the above equation into cell F2:

F2 = 2.155996E-14*(A2)^6 - 6.713456387E-11*(A2)^5 + 8.304701557213E-08*(A2)^4 - 0.000051553921166204*(A2)^3 + 0.0166248198230744*(A2)^2 - 2.52113193385579*(A2) + 142.393374782933

	A	B	C	D	E	F
1		Time	Mean H	RESP	Resp rate	
2	311	00:05:11	121.7236	0.2869	17.214	16.62477
3	312	00:05:12	121.9762	0.2876	17.256	
4	313	00:05:13	122.177	0.2883	17.298	
5	314	00:05:14	122.5045	0.289	17.34	
6	315	00:05:15	122.7572	0.2896	17.376	
7	316	00:05:16	123.0845	0.2903	17.418	
8	317	00:05:17	123.3655	0.291	17.46	
9	318	00:05:18	123.688	0.2916	17.496	
10	319	00:05:19	123.9718	0.2923	17.538	
11	320	00:05:20	124.1961	0.2929	17.574	
12	321	00:05:21	124.3217	0.2935	17.61	
13	322	00:05:22	124.4484	0.2942	17.652	
14	323	00:05:23	124.5145	0.2948	17.688	
15	324	00:05:24	124.5746	0.2954	17.724	
16	325	00:05:25	124.6609	0.296	17.76	
17	326	00:05:26	124.8015	0.2966	17.796	
18	327	00:05:27	124.9284	0.2973	17.838	
19	328	00:05:28	125.0535	0.2978	17.868	
20	329	00:05:29	125.2249	0.2984	17.904	
21	330	00:05:30	125.3898	0.299	17.94	
22	331	00:05:31	125.5344	0.2996	17.976	

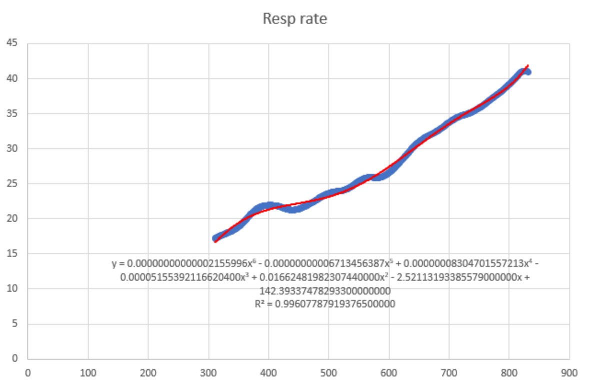

The equation solves the 6th order function for a given "x" which is time in seconds (column A).

Copy F2, select F3 to the end of the F column to populate the cells:

A	B	C	D	E	F
	Time	Mean H	RESP	Resp rate	6th order
311	00:05:11	121.7236	0.2869	17.214	16.62477
312	00:05:12	121.9762	0.2876	17.256	16.71324
313	00:05:13	122.177	0.2883	17.298	16.8008
314	00:05:14	122.5045	0.289	17.34	16.88747
315	00:05:15	122.7572	0.2896	17.376	16.97323
316	00:05:16	123.0845	0.2903	17.418	17.05809
317	00:05:17	123.3655	0.291	17.46	17.14205
318	00:05:18	123.688	0.2916	17.496	17.2251
319	00:05:19	123.9718	0.2923	17.538	17.30725
320	00:05:20	124.1961	0.2929	17.574	17.38851
321	00:05:21	124.3217	0.2935	17.61	17.46886
322	00:05:22	124.4484	0.2942	17.652	17.54831
323	00:05:23	124.5145	0.2948	17.688	17.62686
324	00:05:24	124.5746	0.2954	17.724	17.70451
325	00:05:25	124.6609	0.296	17.76	17.78127
326	00:05:26	124.8015	0.2966	17.796	17.85714
327	00:05:27	124.9284	0.2973	17.838	17.93211
328	00:05:28	125.0535	0.2978	17.868	18.00619
329	00:05:29	125.2249	0.2984	17.904	18.07938
330	00:05:30	125.3898	0.299	17.94	18.15169
331	00:05:31	125.5344	0.2996	17.976	18.22312
332	00:05:32	125.7748	0.3002	18.012	18.29366
333	00:05:33	125.9426	0.3007	18.042	18.36333
334	00:05:34	126.1088	0.3013	18.078	18.43212
335	00:05:35	126.2383	0.3019	18.114	18.50004
336	00:05:36	126.3187	0.3024	18.144	18.5671

Create the first derivitive in column G by just subtracting F3-F2 (based on https://physicslabs.ccnysites.cuny.edu/excel-tips.php):

G3 =SUM(F3,-F2)

	A	B	C	D	E	F	G
1		Time	Mean H	RESP	Resp rate	6th order	First deriv
2	311	00:05:11	121.7236	0.2869	17.214	16.62477	
3	312	00:05:12	121.9762	0.2876	17.256	16.71324	0.088464
4	313	00:05:13	122.177	0.2883	17.298	16.8008	

Copy, paste to extend that operation to the end:

	A	B	C	D	E	F	G
1		Time	Mean H	RESP	Resp rate	6th order	First deriv
2	311	00:05:11	121.7236	0.2869	17.214	16.62477	
3	312	00:05:12	121.9762	0.2876	17.256	16.71324	0.088464
4	313	00:05:13	122.177	0.2883	17.298	16.8008	0.087564
5	314	00:05:14	122.5045	0.289	17.34	16.88747	0.086663
6	315	00:05:15	122.7572	0.2896	17.376	16.97323	0.085762
7	316	00:05:16	123.0845	0.2903	17.418	17.05809	0.08486
8	317	00:05:17	123.3655	0.291	17.46	17.14205	0.083958
9	318	00:05:18	123.688	0.2916	17.496	17.2251	0.083055
10	319	00:05:19	123.9718	0.2923	17.538	17.30725	0.082153
11	320	00:05:20	124.1961	0.2929	17.574	17.38851	0.081252
12	321	00:05:21	124.3217	0.2935	17.61	17.46886	0.080351
13	322	00:05:22	124.4484	0.2942	17.652	17.54831	0.079451
14	323	00:05:23	124.5145	0.2948	17.688	17.62686	0.078552
15	324	00:05:24	124.5746	0.2954	17.724	17.70451	0.077655
16	325	00:05:25	124.6609	0.296	17.76	17.78127	0.076759
17	326	00:05:26	124.8015	0.2966	17.796	17.85714	0.075864
18	327	00:05:27	124.9284	0.2973	17.838	17.93211	0.074972
19	328	00:05:28	125.0535	0.2978	17.868	18.00619	0.074081

Create the second derivitive column in H by subtracting G4-G3:

H4 =SUM(G4,-G3)

	A	B	C	D	E	F	G	H
1		Time	Mean H	RESP	Resp rate	6th order	First deriv	Sec deriv
2	311	00:05:11	121.7236	0.2869	17.214	16.62477		
3	312	00:05:12	121.9762	0.2876	17.256	16.71324	0.088464	
4	313	00:05:13	122.177	0.2883	17.298	16.8008	0.087564	-0.0009
5	314	00:05:14	122.5045	0.289	17.34	16.88747	0.086663	
6	315	00:05:15	122.7572	0.2896	17.376	16.97323	0.085762	

Copy, paste to extend that operation to the end:

A	B	C	D	E	F	G	H
	Time	Mean H	RESP	Resp rate	6th order	First deriv	Sec deriv
311	00:05:11	121.7236	0.2869	17.214	16.62477		
312	00:05:12	121.9762	0.2876	17.256	16.71324	0.088464	
313	00:05:13	122.177	0.2883	17.298	16.8008	0.087564	-0.0009
314	00:05:14	122.5045	0.289	17.34	16.88747	0.086663	-0.0009
315	00:05:15	122.7572	0.2896	17.376	16.97323	0.085762	-0.0009
316	00:05:16	123.0845	0.2903	17.418	17.05809	0.08486	-0.0009
317	00:05:17	123.3655	0.291	17.46	17.14205	0.083958	-0.0009
318	00:05:18	123.688	0.2916	17.496	17.2251	0.083055	-0.0009
319	00:05:19	123.9718	0.2923	17.538	17.30725	0.082153	-0.0009
320	00:05:20	124.1961	0.2929	17.574	17.38851	0.081252	-0.0009
321	00:05:21	124.3217	0.2935	17.61	17.46886	0.080351	-0.0009
322	00:05:22	124.4484	0.2942	17.652	17.54831	0.079451	-0.0009
323	00:05:23	124.5145	0.2948	17.688	17.62686	0.078552	-0.0009
324	00:05:24	124.5746	0.2954	17.724	17.70451	0.077655	-0.0009
325	00:05:25	124.6609	0.296	17.76	17.78127	0.076759	-0.0009
326	00:05:26	124.8015	0.2966	17.796	17.85714	0.075864	-0.00089
327	00:05:27	124.9284	0.2973	17.838	17.93211	0.074972	-0.00089

Plot time (column A) vs second derivitive (column H):

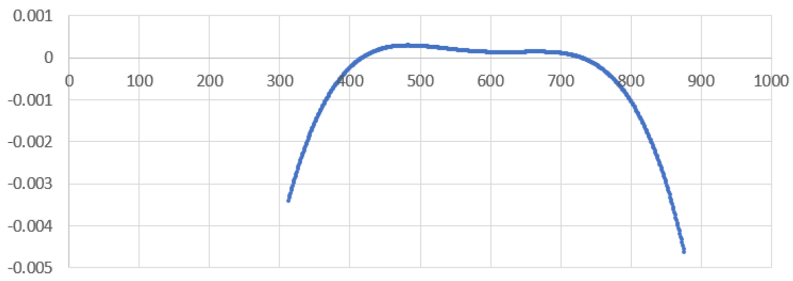

The 2 peaks noted are the maxima, to get the corresponding HR/time inspect the H column:

	A	B	C	D	E	F	G	H	I	J	K	L	M	N	O	P
60	469	00:07:49	146.7644	22.17	22.36056	0.022863	0.00027782									
61	470	00:07:50	146.8978	22.218	22.38371	0.023142	0.00027919									
62	471	00:07:51	147.0237	22.266	22.40713	0.023422	0.00028045									
63	472	00:07:52	147.1641	22.314	22.43083	0.023704	0.00028160									
64	473	00:07:53	147.3098	22.368	22.45482	0.023987	0.00028264									
65	474	00:07:54	147.4723	22.416	22.47909	0.02427	0.00028357									
66	475	00:07:55	147.6015	22.464	22.50364	0.024555	0.00028440									
67	476	00:07:56	147.7718	22.518	22.52848	0.02484	0.00028512									
68	477	00:07:57	147.892	22.566	22.55361	0.025125	0.00028574									
69	478	00:07:58	148.0919	22.62	22.57902	0.025412	0.00028627									
70	479	00:07:59	148.2392	22.674	22.60472	0.025698	0.00028670									
71	480	00:08:00	148.4276	22.722	22.6307	0.025985	0.00028704									
72	481	00:08:01	148.6293	22.776	22.65698	0.026273	0.00028729									
73	482	00:08:02	148.8357	22.83	22.68354	0.02656	0.00028745									
74	483	00:08:03	149.0562	22.884	22.71038	0.026848	0.00028752									
75	484	00:08:04	149.2575	22.944	22.73752	0.027135	0.00028751									
76	485	00:08:05	149.4433	22.998	22.76494	0.027423	0.00028741									
77	486	00:08:06	149.655	23.052	22.79265	0.02771	0.00028724									
78	487	00:08:07	149.9058	23.106	22.82065	0.027997	0.00028699									
79	488	00:08:08	150.0571	23.16	22.84893	0.028284	0.00028666									
80	489	00:08:09	150.2945	23.208	22.8775	0.02857	0.00028626									
81	490	00:08:10	150.4883	23.256	22.90636	0.028856	0.00028578									
82	491	00:08:11	150.7155	23.304	22.9355	0.029141	0.00028524									
83	492	00:08:12	150.9938	23.346	22.96492	0.029425	0.00028463									

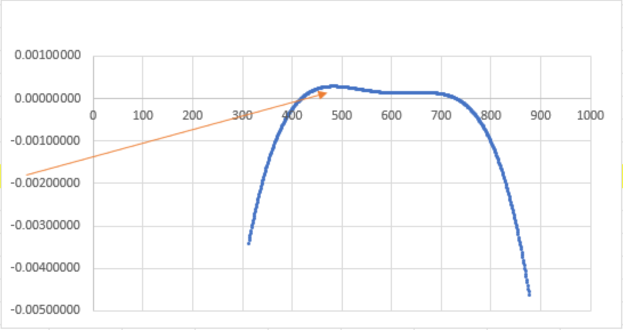

The above is the first maxima at 483 seconds, 149 bpm.

And the second maxima (always inspect the numerical data, it may be subtle):

50	659	00:10:59	169.625	31.554	30.75238	0.058977	0.00013692
51	660	00:11:00	169.801	31.602	30.81149	0.059114	0.00013709
52	661	00:11:01	169.9668	31.65	30.87074	0.059251	0.00013724
53	662	00:11:02	170.1329	31.698	30.93013	0.059389	0.00013737
54	663	00:11:03	170.3021	31.74	30.98966	0.059526	0.00013747
55	664	00:11:04	170.419	31.782	31.04932	0.059664	0.00013755
56	665	00:11:05	170.5608	31.824	31.10912	0.059801	0.00013761
57	666	00:11:06	170.6911	31.86	31.16906	0.059939	0.00013763
58	667	00:11:07	170.8225	31.902	31.22914	0.060077	0.00013763
59	668	00:11:08	170.926	31.944	31.28935	0.060214	0.00013759
60	669	00:11:09	171.0198	31.986	31.34971	0.060352	0.00013753
61	670	00:11:10	171.1167	32.028	31.41019	0.060489	0.00013743
62	671	00:11:11	171.2109	32.076	31.47082	0.060626	0.00013729
63	672	00:11:12	171.2876	32.118	31.53158	0.060764	0.00013712
64	673	00:11:13	171.4084	32.166	31.59248	0.0609	0.00013690
65	674	00:11:14	171.4192	32.214	31.65352	0.061037	0.00013665
66	675	00:11:15	171.5444	32.262	31.7147	0.061173	0.00013636
67	676	00:11:16	171.6503	32.31	31.776	0.061309	0.00013602
68	677	00:11:17	171.7256	32.358	31.83745	0.061445	0.00013563
69	678	00:11:18	171.8513	32.412	31.89903	0.06158	0.00013520

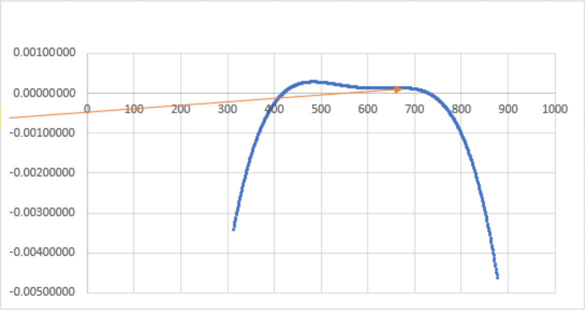

The above is the second maxima at 666 seconds, 170 bpm.
